# Blood product use for radiological/nuclear trauma: product development and US regulatory considerations

**DOI:** 10.1136/tsaco-2023-001123

**Published:** 2024-01-05

**Authors:** Toby A Silverman, Annette M Shadiack, Kimberly A Hofmeyer, Ashley E Cecere, Derek L Eisnor, Corey M Hoffman, Shannon G Loelius, Aditiben Patel, Mary J Homer

**Affiliations:** 1 Tunnell Government Services, Bethesda, Maryland, USA; 2 Division of Chemical, Biological, Radiological, and Nuclear Medical Countermeasure, Biomedical Advanced Research and Development Authority, Washington, District of Columbia, USA; 3 Biomedical Advanced Research and Development Authority, Washington, District of Columbia, USA; 4 Division of Clinical Development, Biomedical Advanced Research and Development Authority, Washington, District of Columbia, USA; 5 Division of Regulatory and Quality Affairs, Biomedical Advanced Research and Development Authority, Washington, District of Columbia, USA

**Keywords:** blood, hemorrhage, biomedical research, policy

## Abstract

Blood products are likely to be critical components of the medical response to nuclear detonation, as the hematopoietic subsyndrome of acute radiation syndrome (H-ARS) includes depletion of platelets and red blood cells that can lead to lethal hemorrhage and anemia. There is, however, only limited clinical information on the use of blood products to treat H-ARS. As currently configured, the US blood supply cannot meet the predicted surge in blood product demand that is likely to occur short-term and possibly long-term in the event of a large nuclear detonation. As part of the Administration for Strategic Preparedness and Response within the US Department of Health and Human Services, the Biomedical Advanced Research and Development Authority (BARDA) is addressing this preparedness gap by supporting the development of novel blood products and devices with characteristics that improve blood product storage and use in austere operational environments. The US Food and Drug Administration’s Center for Drug Evaluation and Research (CDER) recently issued draft guidance on the development of drugs and biologics regulated by CDER to prevent or treat Acute Radiation Syndrome under the provisions of the “Animal Rule.” The commentary provided here discusses the unique regulatory scheme for transfusion components and blood products regulated as biological drugs by Center for Biologics Evaluation and Research, including the ambiguity surrounding the evidentiary requirements for their approval for H-ARS, and whether, under certain circumstances, a specific H-ARS indication is necessary if relevant commercial indications are approved.

## Introduction

**Figure 1:**
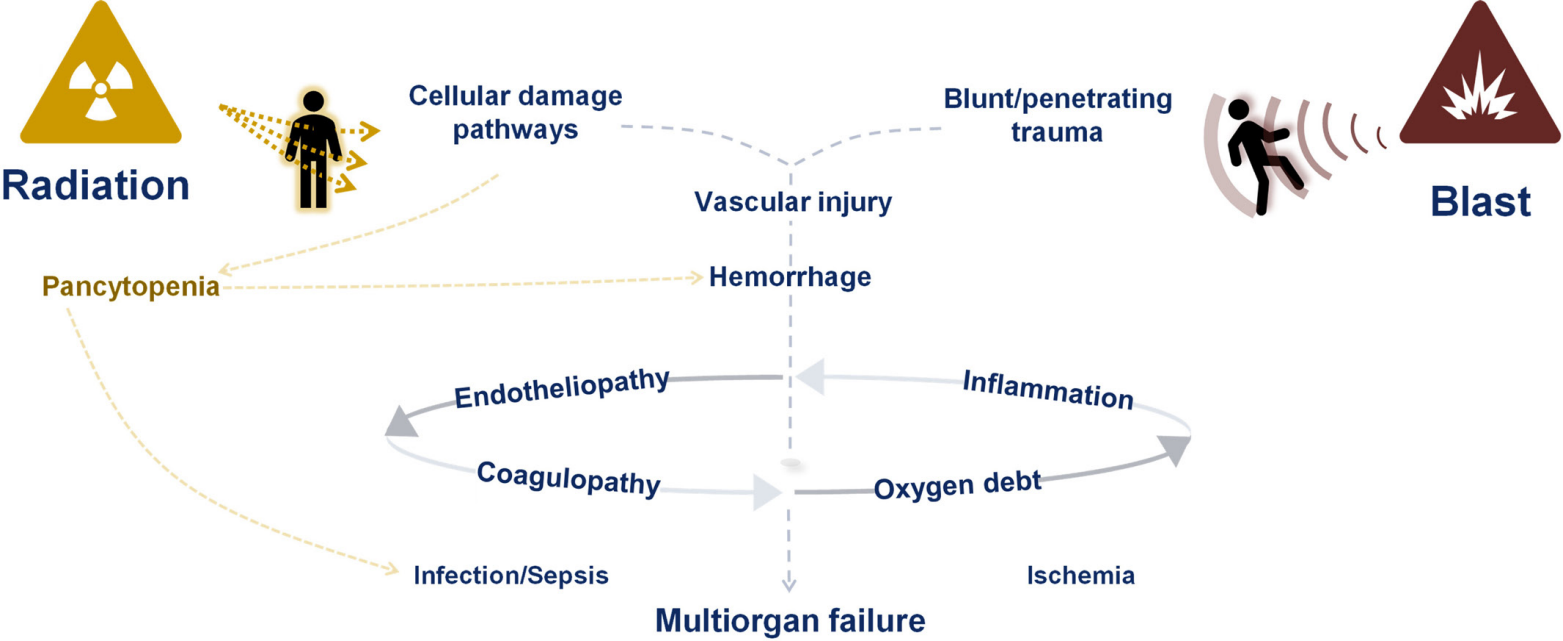
Features thought to be common to radiation injury and mechanical trauma include vascular injury, hemorrhage, endotheliopathy, coagulopathy, and inflammation. These may lead to eventual multiorgan failure.

Nuclear detonation modeling predicts casualties in the hundreds of thousands with the injury type and severity dependent on detonation yield, height of burst, population density, building types, shielding, and weather patterns.^1^ The medical consequences of nuclear detonation include an array of traumas including radiation injury, blast injury (mechanical trauma), and burns. The hematopoietic subsyndrome of acute radiation syndrome (H-ARS) is characterized by neutropenia, thrombocytopenia, and anemia. In addition, the interplay between inflammation and coagulation plays a significant role in radiation injury and death.^2^ There is significant overlap in the downstream pathophysiologic effects of radiation injury and mechanical trauma, including the development of vascular/endothelial injury, coagulopathic changes, multiorgan failure, and death. Features thought to be common to both radiation injury and trauma are summarized in figure 1.

At present, the clinical management of H-ARS focuses on restoring depleted hematopoietic cell populations and addressing potentially lethal infections, hemorrhage, and anemia. Future interventions may address the inflammatory and endothelial effects of acute radiation and may also be beneficial in mitigating multiorgan failure/early death, and the delayed effects of radiation injury.^3^

Blood products may be an important component of the medical response to nuclear detonation and are recommended to treat H-ARS thrombocytopenia and anemia, although information on the use of blood products to treat ARS is very limited.^4^ The need for blood products after a nuclear detonation is predicted to greatly exceed availability, with gaps of up to 147 000 red blood cell (RBC) units and 5.7 million platelet units predicted for a 10-kiloton maximum detonation scenario in Washington, DC.^5^ However, the capacity to address these gaps is limited. As currently configured, the US blood supply system cannot adequately provide surge capacity to collect and distribute blood products in response to a nuclear mass casualty incident (MCI).^6^ Resupply in this scenario may be impacted by a limited donor pool and by blood type matching requirements, whereas stockpiling and field care use may be logistically challenging due in part to cold chain reliance, limited product shelf life, and bulky packaging.

Within the Administration for Strategic Preparedness and Response (ASPR), the BARDA Division of Chemical, Biological, Radiological and Nuclear (CBRN) Medical Countermeasures (MCMs) is addressing these gaps by supporting the development of blood products that have improved physiological, storage, and distribution characteristics. The Radiological and Nuclear Countermeasures (R/N) Branch of CBRN focuses on the development of products and devices that facilitate stockpiling, prepositioning, deploying, and using H-ARS treatments in the austere field and hospital environments anticipated during a nuclear MCI.^7^ Such product development programs must consider the unique regulatory environment for blood products.

Blood products are regulated by the US Food and Drug Administration (FDA) through application of manufacturing standards and current good manufacturing practice (cGMP) requirements that ensure compliance with standards. The extent to which a novel blood product deviates from these accepted standards will inform the evidence needed to support FDA approval. The ambiguity surrounding evidentiary requirements for approval of blood products raises questions about whether an H-ARS indication is necessary when the product has an approved commercial indication. These questions are examined here through a review of the history of blood products and biologics, relevant FDA regulations, and past and present development programs.

## When an H-ARS specific indication may be necessary

### Blood product regulatory background

Development of blood products to treat H-ARS must be built on a clear understanding of regulatory terminology that is codified in the US Code of Federal Regulations (CFR). “Blood” means a product that is a fluid containing dissolved and suspended elements which was collected from the vascular system of a human.^8^ “Blood component” means a product containing a part of blood separated by physical or mechanical means.^8 9^ “Blood product’” means a product that consists of human whole blood, plasma, serum, or any derivative products.^9^ Blood products may include those that meet the definition of a device under the US Federal Food, Drug, and Cosmetic (FD&C) Act and those that would be licensed as a biological drug product. Per regulatory definition, blood products therefore include both blood and blood components.^9 10^

Blood products are enumerated as biologics under the Public Health Service (PHS) Act and are licensed under Section 351 of this Act in accordance with FDA standards for safety, purity, and potency for both the product and the processing or manufacturing facility.^10^ Blood products are also subject to provisions under the FD&C Act as discussed in part below.^11^

### Approval process elements for blood products and cellular products

#### Registration and listing

Blood products have some unique licensing and labeling elements compared with other biologics because their supply is managed by a network of independent facilities all processing the same starting materials (ie, blood and blood components) and manufacturing largely the same products. The FDA regulates blood products through the development and application of standards with which these independent manufacturers and distributers must comply.^9 12–14^ Any establishment that processes and supplies blood products is required to register with the FDA and list all blood products in commercial distribution on an annual basis and is subject to FDA inspection for cGMP standard adherence.^8 15 16^

#### Licensure

Licensure of blood products affects whether interstate distribution and commerce may occur. Licensed blood and blood products that are regulated under Section 351 of the PHS Act may be distributed in interstate commerce.^10^ Blood and blood products that are produced and distributed solely within a state (intrastate commerce) are not subject to the licensing provisions under Section 351. However, they are subject to PHS Act Section 361 that includes regulations to control communicable diseases.^17^

#### Deviations from cGMP

Deviation from the applicable standards requires prior approval by the FDA either in an amendment to the license of an establishment or in the form of a variance under 21 CFR §640.120, which defines exceptions and alternative procedures to requirements regarding blood, blood components, and blood products.^18 19^

#### Labeling

Blood product cGMP standards also dictate requirements for container labeling, which include the license number permitting interstate commerce.^20^ Blood product labels are affixed to the product container, which limits space for detailed information. To account for this and provide adequate use directions, FDA requires a circular of information as an extension of blood container labeling.^21^ Individual facilities may develop their own circular with FDA approval as long as the specific procedures are consistent with and meet the requirements contained in regulations. Alternatively, facilities may use the Circular of Information (the Circular) that is developed and published by the Association for the Advancement of Blood and Biotherapies (AABB). Any update to the Circular is reviewed by FDA and once accepted, is announced in FDA guidance and published by AABB as an updated Circular.^22^

The Circular enables standardization of production and provides general guidance for blood product use. Blood components listed in the current Circular include RBC Components, Plasma Components, Cryoprecipitated Components, Platelet Components, and Granulocyte Components.^19^ The Circular describes the actions, indications, contraindications, dosage and administration, and side effects and hazards of blood and blood components, the preparation of subtypes of blood components, as well as further processing of blood components (eg, pathogen reduction or leukocyte reduction). Additional components or “further processing” procedures may be added as existing device manufacturers add to or update the device instructions for use, as variances are granted, or as new devices are approved.

#### Manufactured blood products and cellular therapies

Blood and blood components can be further manufactured to produce analogs of blood components, here referred to as “manufactured blood products.” Blood and blood components intended for further manufacture are regulated by FDA under various provisions captured under 21 CFR §600-640.^8 12^ Manufactured blood products are subject to the requirements for demonstration of substantial evidence of effectiveness as demonstrated through the conduct of adequate and well-controlled clinical trials in line with other biologics that are not conventional blood transfusion products. Individual labeling of such products must comport with the labeling requirements for drugs and biologics. Examples of manufactured blood products include clotting factor concentrates and immune globulin products that are purified from plasma, recombinant analogs, etc.

Manufactured cell products also can be produced from other starting materials such as stem cells. Manufactured blood products developed from stem cells have specialized considerations in addition to the characterization requirements for human blood-derived products. Human cells, tissues, and cellular and tissue-based products (HCT/Ps) are defined as articles containing or consisting of human cells or tissues that are intended for implantation, transplantation, infusion, or transfer into a human recipient.^23 24^ Like blood and blood components, HCT/Ps may be regulated solely under provisions of PHS Act Section 361 (regulations to control communicable diseases) or may be subject to regulation under PHS Act Section 351 (regulation of biological products). Regulations captured under 21 CFR §1271 address the distinction between HCT/Ps that are Section 361 versus Section 351 products.^23^ Under a tiered, risk-based approach, HCT/Ps that meet specific criteria or fall within detailed exceptions do not require premarket review and approval. In general, manufactured cell products reviewed under Section 351 of the PHS Act will require demonstration of safety and efficacy through the conduct of adequate and well-controlled clinical trials and will be reviewed in the Center for Biologics Evaluation and Research (CBER) by the Office of Tissues and Advanced Therapies with input from the Office of Blood Research and Review. At present, there are no approved stem-cell derived blood products in the USA. Stem cell-derived RBCs have been developed and are under study in the UK (RESTORE (Recovery and survival of stem cell originated red cells; ISRCTN42886452, https://www.nhsbt.nhs.uk/clinical-trials-unit/current-trials-and-studies/restore/)).

Considerations related to the regulation of HCT/Ps are captured in 2020 FDA Guidance entitled “Regulatory Considerations for Human Cells, Tissues, and Cellular and Tissue-Based Products: Minimal Manipulation and Homologous Use Guidance for Industry and Food and Drug Administration Staff.”^25^ According to these criteria, stem cell-based products would be regulated under Section 351 of the PHS Act and be subject to the requirements in the FD&C Act and the PHS Act. As noted, these requirements would include the demonstration of substantial evidence of effectiveness (drugs and biologics) or valid scientific evidence (devices).

### Using blood products for H-ARS

The BARDA R/N Branch supports advanced development of novel blood products with improved performance or storage characteristics over conventional blood products. Improved characteristics address the logistical challenges of conventional blood products that limit their use far forward in emergency responses while maintaining critical functionality. These may include reduced cold chain reliance and improved shelf life, stability, and ease of use. Program goals include FDA approval of relevant commercial indications as well as use of the novel blood products to address H-ARS and/or traumatic injury. Whether an H-ARS-specific indication is necessary in addition to the intended commercial indication(s) is better understood through the examples given below that highlight the implications of the regulatory framework for blood products summarized above.

#### When additional studies to support an indication for H-ARS may not be needed

Conventional platelets, plasma, and RBCs do not carry a specific label for use in the prevention or treatment of H-ARS. However, the indication statements for these blood components in the Circular are sufficiently broad that they would appear to encompass use for the prevention or treatment of H-ARS; accordingly, it is unlikely that label modifications would be necessary.^19^ In line with this concept, current medical guidelines for the transfusion of blood components for H-ARS use evidence-based thresholds that are comparable to the indications, dose, and administration instructions in the Circular.^14^ The Circular is also cited as a general reference for transfusion guidelines by the Radiation Emergency Medical Management (REMM) Website of the US Department of Health and Human Services (HHS).^26^

Psoralen-based pathogen reduction technology for platelets and plasma (Cerus Corporation, Concord, CA) was approved by the FDA in 2014.^27 28^ The evidence leading to FDA approval for the device was derived from clinical trials performed by the commercial sponsor to demonstrate the safety and efficacy of the pathogen-reduced products for the same indications carried by conventional platelets and plasma as described in the Circular. Labeling for the approved medical device contains language related to the intended use of the device as well as instructions for use. The pathogen-reduced blood products produced in a distributed manner by blood centers using the approved device are covered under the section of the Circular for the corresponding conventional blood product. However, licensed blood centers that want to use the pathogen reduction device are required to amend their licenses and receive approval from FDA for the change to implement the technology and to distribute the pathogen-reduced blood components in interstate commerce.^29^ Since these pathogen-reduced blood products are subject to instructions in the Circular, like the conventional blood product example above, clinical trials to evaluate use of pathogen-reduced platelets to address H-ARS apparently would not be required.

#### When additional studies to support an indication for H-ARS may be needed

Octaplas (Octapharma, Paramus, NJ), on the contrary, is an example of a manufactured blood product. Octaplas is a sterile, pyrogen-free, frozen solution of solvent/detergent-treated pooled human plasma.^30^ The concentrations of components of Octaplas are comparable to reference ranges for healthy blood donors, and the mechanism of action is replacement of human plasma proteins. Octaplas was subject to the requirements for adequate and well-controlled clinical trials to demonstrate efficacy and safety for the labeled indications, which include replacement of multiple coagulation factors in patients with acquired deficiencies due to liver disease or undergoing cardiac operation or liver transplantation, and for plasma exchange in patients with thrombotic thrombocytopenic purpura. These indications represent a subset of the stated indications in the Circular for plasma for transfusion. Additional label indications would be subject to the same requirement for adequate and well-controlled clinical trials.

Worldwide, international versions of Octaplas in Europe, Australia and Canada, respectively, Octaplas LG (Octapharma, Manchester, UK) and Octaplasma (Octapharma, Toronto, Canada)are approved for additional indications; specified conditions of use differ in the various venues where the product is approved.^31 32^ A recent comparison of a lyophilized formulation of Octaplas LG to the frozen version of Octaplas LG has been performed and shows that frozen and lyophilized versions demonstrate comparable physicochemical properties when thawed or reconstituted. Both products were also comparable to fresh frozen plasma (FFP) with the exception of protein S and plasmin inhibitor levels which were lower than in FFP.^33^ Recently, a lyophilized version of Octaplas LG has been approved by EMA for prehospital use.^34^ In the USA, what remains unclear is whether adequate and well-controlled clinical trials will be required for the manufacturer to demonstrate comparability of the lyophilized presentation to the currently licensed product.

#### Case study: dried plasma products

Dried plasma products may be prepared either in a distributed manner at blood centers using devices designed for the local preparation of dried plasma or manufactured by a commercial sponsor in interstate commerce. FDA has provided guidance on the clinical evidence standard for approval of dried plasma products for use when conventional plasma for transfusion is not available.^35^ The guidance directs companies developing dried plasma-generating devices to include the necessary equipment with precise specifications for a blood establishment to process the dried plasma product.^35^ Recommendations for the design of clinical trials to demonstrate that dried plasma products may be used when conventional plasma is available have not been provided.

The difference in regulatory pathways for distributed versus centralized manufacture of blood products highlights additional considerations related to whether specific studies in H-ARS are needed. In the instance of distributed manufacture by blood centers, a dried plasma product would be made at a blood center using a device approved/cleared by FDA for preparation of dried plasma. Responsibility for demonstration of efficacy and safety of the dried plasma product made by the device would rest with the device manufacturer, and device labeling would provide appropriate instructions for use. However, once approved by the FDA, the next iteration of the Circular likely would incorporate instructions for further processing of dried plasma with the proviso that use is limited to situations where conventional plasma is not available. An update to the Circular to include use when conventional plasma is available would not occur until the device manufacturer had conducted appropriate studies. The design of these studies would be the subject of negotiations with FDA. On approval of the device, licensed blood centers could amend their licenses to permit deployment of the device and shipment of the dried plasma product in interstate commerce. This regulatory pathway is similar to that described for the Cerus pathogen reduction device and may not require a specific H-ARS indication.

In the instance of central manufacture by a commercial sponsor, a dried plasma product would be regulated as a manufactured blood product and not as blood or a blood component. This is because the manufacturing process would involve more than physical or mechanical separation and final manufacture would not occur at a blood center. The commercial sponsor manufacturing the dried plasma product would be required to conduct adequate and well-controlled clinical trials to support product labeling. Labeling will be unique to the specific product and would not be added to the Circular.

Use of manufactured blood products for the prevention or treatment of H-ARS would be considered off-label even if the manufactured product could be used in lieu of the blood component for all the indications related to that blood component. Therefore, manufactured blood products would most likely require additional studies and a label change specific to H-ARS. This situation would be similar to that of products such as the colony stimulating factors where studies under the Animal Rule were required to develop the specific label indication for H-ARS.^36^

#### Off-label use of blood products

Off-label use of an approved product when the intent is the “practice of medicine” does not require the submission of an Investigational New Drug Application, Investigational Device Exemption, or review by an Institutional Review Board.^37^ However, the manufacturer would not be able to promote use for H-ARS unless appropriate studies had been performed. For example, studies were required for the approval of colony stimulating factors and a thrombopoietin receptor agonist treatment for H-ARS even though the drugs were previously approved and marketed for the treatment of neutropenia and thrombocytopenia, respectively, for other commercial indications.

There is precedent for the US government (USG) making off-label MCM use recommendations for products with an approved commercial indication. In 2004 prior to the approval of colony stimulating factors for H-ARS, the Strategic National Stockpile (SNS) Radiation Working Group published guidelines for the medical management of H-ARS based on the summation of evidence from clinical use in approved indications and animal studies in H-ARS models.^38^ In addition, guidelines for off-label use of treatments for nerve agent poisoning when approved MCMs are not available are published on the US Department of Health and Human Services’ Chemical Hazards Emergency Medical Management (CHEMM) Web site. The guidelines for nerve agent contingency MCMs were developed by the Public Health Emergency Medical Countermeasures Enterprise (PHEMCE) Chemical Integrated Program Team (Chem IPT).^39 40^

Off-label use of a manufactured blood product for ARS may also be possible under Emergency Use Authorization (EUA); however, such authorization would require a declared emergency for a threat that results in serious or life-threatening conditions with no adequate alternatives to the product.^41^

## Developing (blood) products for H-ARS

### Program requirements and assumptions

CBRN product development programs must consider strategies to maximize the likelihood of regulatory success and the sustainability of the approved product. This is important to ensure that products remain viable and, in turn, available when a need arises.

One successful strategy used by BARDA is the label expansion of commercially marketed products for an H-ARS indication. Label expansion, or drug repurposing, can reduce development costs, efforts, and timelines. This approach also provides the opportunity to leverage existing dosing parameters, drug activity, and safety data to inform H-ARS dose selection, particularly if the mechanism of action for the approved indication is closely aligned with what is needed for use in H-ARS. The five MCMs currently approved for H-ARS are label expansions for products with mechanisms of action and indications that had a clear benefit for H-ARS.

The Neupogen (Amgen, Thousand Oaks, CA), Neulasta (Amgen, Thousand Oaks, CA), Leukine (Partner Therapeutics, Lexington, MA), and Udenyca (a biosimilar to Neulasta; Coherus Biosciences, Redwood City, CA) colony stimulating factors were initially approved to address neutropenia in patients with cancer receiving specific therapies.^42–44^ Nplate (Amgen, Thousand Oaks, CA), a thrombopoietin receptor agonist, was initially approved to treat thrombocytopenia in patients with immune thrombocytopenia.^45^ The original clinical indications for the five approved drug products were assessed by FDA to be insufficient to support an indication for H-ARS in the absence of data on effect after lethal doses of radiation. As noted above, substantial evidence of effectiveness as demonstrated by adequate and well-controlled clinical trials is required for manufactured blood products subject to Section 351 of the PHS Act and for drugs subject to section 505 of the Food Drug and Cosmetic Act. Because it is not ethical to expose humans to lethal doses of radiation, development of such products is covered by provisions of the Animal Rule. Recently, FDA’s Center for Drug Evaluation and Research (CDER) issued draft guidance on the development of drug and biological drug products regulated by CDER to treat or prevent H-ARS using the Animal Rule.^46^ That guidance summarizes the approach CDER took in approving the five drug products and highlights some of the issues associated with using the Animal Rule to support development of products for H-ARS.

For investigational stage products without an approved commercial indication, a risk reduction strategy is concurrent pursuit of a commercial indication in addition to an H-ARS indication. Although this may increase development cost and timeline, this strategy has several benefits: (1) leverages human safety and efficacy data across the H-ARS and commercial indications; (2) reduces the need for USG procurement by allowing for vendor-managed inventory; (3) enables positioning of products closer to the point of need for more rapid deployment; and (4) increases the market size to support a more sustainable business model for the developer.^47 48^

Alternative development strategies also exist where a product is not developed with the intent of approval for both a commercial and H-ARS indication. Five example development strategies are described below (table 1). These scenarios encompass development of drug and biologic products in addition to blood, blood components, and blood products.

**Table 1:** Development program strategies for H-ARS MCMs

Strategy	Label indication		Requires nonclinical H-ARS studies	Description
	Commercial	H-ARS		
1	–	✓	✓	Development under Animal Rule for H-ARS indication based on pivotal evidence of efficacy in nonclinical models Safety trials performed in healthy human subjects
2	✓	–	–	Approval for commercial indication supported by human trials that demonstrate safety and pivotal evidence of efficacy No nonclinical studies specifically addressing H-ARS
3	✓	–	✓	Approval for commercial indication that is likely to translate to benefit for H-ARS Nonclinical studies addressing H-ARS performed and available for EUA submission
4	✓	✓	✓	Approval for commercial indication that works via mechanism of action that is relevant to that required to address H-ARS Concurrent nonclinical efficacy studies for H-ARS indication for approval under the Animal Rule
5	✓	✓	✓	Existing approval for commercial indication that works via mechanism of action that is relevant to that required to address H-ARS Subsequent nonclinical efficacy studies for addition of H-ARS indication approved under the Animal Rule

EUA, Emergency Use Authorization; H-ARS, hematopoietic subsyndrome of acute radiation syndrome; MCMs, Medical Countermeasures .

#### Strategy 1

Only pursuing an indication for H-ARS provides an opportunity to evaluate products that would otherwise not exist for serious or life-threatening conditions. However, such MCMs tend to have limited markets as the USG is more likely to be the only purchaser. This is a risk to the government and may affect company risk if USG procurement alone is not enough to sustain the business financially. This strategy does not appear to be a viable option for most blood products.

#### Strategy 2

Only pursuing a commercial indication is clearly beneficial for novel blood products that are expected to be manufactured by blood centers and described in the Circular. The product probably would not need an H-ARS-specific indication but would be used in accordance with the stated indications in the Circular.

#### Strategy 3

Manufactured blood products, which will *not* be included in the Circular, will be governed by their own specific label. The risk of the product not being effective for H-ARS could be reduced by conducting nonclinical studies in H-ARS models in addition to pursuing a related commercial indication even if approval for the H-ARS indication is not sought.

This strategy was followed in 2004 when the SNS Radiation Working Group published guidelines for the medical management of H-ARS using colony stimulating factors based on the summation of evidence from clinical use and animal studies prior to their approval under Animal Rule in 2014.^38^ This strategy is more proactive than strategy 2, in that the addition of the nonclinical data better positions the product for issuance of an EUA in a declared emergency with an unmet medical need due to limited availability of conventional products.^41^ The primary challenge of an EUA is that the specific evidentiary standards required from H-ARS nonclinical studies may not be known in advance without prior feedback from the FDA clarifying the evidentiary standards.

#### Strategy 4

Pursuit and approval of both commercial and H-ARS indications in parallel would result in higher program costs and longer timelines but has an advantage for new products. The H-ARS indication will enable efficient use during an emergency, whereas the commercial indication will help secure an outside market. When considering this strategy, an important factor to consider is prioritizing the commercial indication along with frontloading key commercial clinical program milestones to maximize program success and achieve the desired approvals while minimizing program risks. If the commercial indication fails, continued development of the product for H-ARS alone incurs the same risks as strategy 1.

#### Strategy 5

Label expansion of commercially marketed products for H-ARS is beneficial in reducing the cost, effort, and time needed for development. To take advantage of these benefits, the mechanism of action of the approved commercial indication should be relevant to H-ARS so that the existing information about dosing parameters, activity, and real-world safety can guide the H-ARS indication development.

### Commercial and H-ARS indications for investigational stage products

Products currently approved under the Animal Rule include novel products that either only have a CBRN indication due to their specificity or are label expansion products previously approved for commercial indications.^49^ Manufactured blood products seeking an H-ARS indication, like any MCM, could be approved under the Animal Rule regulatory pathway as radiation injury is a serious or life-threatening indication where it is not ethical or feasible to conduct human efficacy studies.^36^ The Animal Rule allows for adequate and well-controlled animal efficacy trials to provide the pivotal evidence of efficacy for the threat indication in lieu of human efficacy trials; however, human safety trials are still required.^36^ The FDA will rely on evidence from animal studies to support approval under the Animal Rule when the following criteria are met^36^:

1. There is a reasonably well-understood mechanism for the toxicity of the agent and its amelioration or prevention by the product.
2. The effect is demonstrated in more than one animal species expected to react with a response predictive for humans, unless the effect is demonstrated in a single animal species that represents a sufficiently well-characterized animal model for predicting the response in humans;
3. The animal study endpoint is clearly related to the desired benefit in humans—generally the enhancement of survival or prevention of major morbidity.
4. The data or information on the kinetics and pharmacodynamics of the product or other relevant data or information, in animals or humans, allow selection of an effective dose in humans.

#### USG Priorities for blood product development

Government agencies with a mission to address preparedness goals and improve the ability to treat hemostatic dysregulation injuries worked together to develop and implement an interagency strategic plan. This strategic plan identified a critical need for improved capabilities to understand, detect, prevent, mitigate, treat, and recover from the pathophysiologies that occur along the continuum from blood-vascular injury to multiorgan failure, regardless of the inciting insult. Blood-vascular injury is an underlying cause for many types of injuries and disease, including trauma, radiation injury, select infectious diseases, and sepsis. New technologies that are focused on preventing or addressing the underlying pathophysiological mechanisms of blood-vascular injury, and the resulting disorders of metabolism, are anticipated to enhance USG medical preparedness across operational domains and threat spaces.

Specifically, the ASPR/BARDA portfolio should include programs that could enhance our ability to respond to a MCI. Several characteristics of blood products and the blood product industry need consideration when preparing for a sudden (no-notice), high-consequence, MCI Current blood products for infusion are complex, labile, donor-derived fluids or tissue components with relatively resource-intensive storage and utilization requirements. Given these considerations, the best means of provision is to have easy-to-prepare products that can be kept in readily available commercial or end-user inventories managed to avoid expiration. The potential size of such managed inventories is driven primarily by shelf-life and by the rate of conventional use. The current blood product focus areas delineated in the ASPR/BARDA Broad Agency Announcement include:

1. Technologies that improve the safety and availability of blood products in a mass casualty event;
2. Pharmaceuticals that could be used in lieu of blood products to treat hemorrhage.
3. Next generation blood products derived from stem and progenitor cells to produce red cell, platelet, or white blood cell products.
4. Technologies for reliably producing hematopoietic stem cells and their progenitors, including optimization of directed differentiation and engraftment of functional and safe hematopoietic cells.

## Conclusion

This review has highlighted some of the significant and unique challenges for the development of novel blood products including for use in the context of a nuclear detonation. The decision whether to pursue evaluation for H-ARS under the Animal Rule may be impacted by the regulatory pathway under which a product is evaluated. In the end, the determination is based on product-specific characteristics and should be informed by a full understanding of the risks and benefits of the product. Early and frequent engagement by product development sponsors with the FDA is needed to ensure a viable development strategy and final approved product that can be deployed readily.
